# Assessment of the impact of reopening strategies on the spatial transmission risk of COVID-19 based on a data-driven transmission model

**DOI:** 10.1038/s41598-023-37297-5

**Published:** 2023-07-10

**Authors:** Jing Wang, YuHui Huang, Ying Dong, BingYing Wu

**Affiliations:** 1 School of Economics and Management, Fuzhou University, Fuzhou, 350116 China; 2 Emergency Management Research Center, Fuzhou University, Fuzhou, 350116 China

**Keywords:** Scientific data, Risk factors

## Abstract

COVID-19 has dramatically changed people's mobility geste patterns and affected the operations of different functional spots. In the environment of the successful reopening of countries around the world since 2022, it's pivotal to understand whether the reopening of different types of locales poses a threat of wide epidemic transmission. In this paper, by establishing an epidemiological model based on mobile network data, combining the data handed by the Safegraph website, and taking into account the crowd inflow characteristics and the changes of susceptible and latent populations, the trends of the number of crowd visits and the number of epidemic infections at different functional points of interest after the perpetration of continuing strategies were simulated. The model was also validated with daily new cases in ten metropolitan areas in the United States from March to May 2020, and the results showed that the model fitted the evolutionary trend of realistic data more accurately. Further, the points of interest were classified into risk levels, and the corresponding reopening minimum standard prevention and control measures were proposed to be implemented according to different risk levels. The results showed that restaurants and gyms became high-risk points of interest after the perpetration of the continuing strategy, especially the general dine-in restaurants were at higher risk levels. Religious exertion centers were the points of interest with the loftiest average infection rates after the perpetration of the continuing strategy. Points of interest such as convenience stores, large shopping malls, and pharmacies were at a lower risk for outbreak impact after the continuing strategy was enforced. Based on this, continuing forestallment and control strategies for different functional points of interest are proposed to provide decision support for the development of precise forestallment and control measures for different spots.

## Introduction

COVID-19 as a new viral pattern with sudden, epidemic, and global nature has made everyone pay a heavy price for it^1^. As of January 11, 2023, the cumulative number of diagnosed cases worldwide exceeds 660 million, and the pandemic might have taken upwards of 18 million lives^2^. Covid-19 is the second major public health emergency since the SARS outbreak, and the lives of people around the world are seriously threatened, the impact involves economic, political, and cultural aspects, and all regions of the world have suffered incalculable losses.

But recently surveillance and reporting of virus movement has begun to slow down in countries around the world. Governments ended lockdowns, reopened schools, and scaled back or abandoned mask-wearing mandates^3^. Countries worldwide are slowly rolling out their business reopening plans. But this comes at a time when the highly infectious subvariant of Omicron is spreading around the world and case rates and hospitalizations are picking up. So this is no time to stop tracking COVID-19^4^. The reopening policy may expose the public to the risk of new COVID-19 infections^5^. However, due to natural and social factors, the evolutionary characteristics of the epidemic vary in different spaces^6^. In large cities, the epidemic spreads in a short time due to the concentration of the population and the mobility of people. In contrast, in remote rural areas, where the population is small and people are not closely connected, it takes a longer time for the virus to spread^7^. Therefore, according to the inherent characteristics of different regions, we should not adopt a "one-size-fits-all" approach when formulating prevention and control measures, but should understand and grasp the law of transmission and then formulate scientific and reasonable countermeasures in a "localized", precise and effective manner.

Relevant studies have shown that measures such as detection and tracking, banning large gatherings, closing nonessential businesses and schools and universities, limiting international and domestic mobility, and physical isolation are essential to delay and contain the COVID-19 pandemic^8^. In addition, public spaces with personal "stay" characteristics have a greater impact on COVID-19 transmission^9^. Assessing the impact of these sites on COVID-19 transmission is important for determining the type of public space at risk of transmission. Decision-making and evaluation or interventions during the pandemic require not only specific, reliable, and timely data on infection but also on human behavior, especially on mobility and physical activity. The correct and prudent use of mobile network data will be an important arsenal of tools to support public health actions during the COVID-19 pandemic.

The chain of epidemic transmission is highly variable across countries, regions, and even communities, and therefore the "social universality" of epidemic prevention and control measures is not scientifically sound, rational, or referable. Modeling and analyzing the transmission process of epidemic infectious diseases through empirical data can help to deeply explore the inner transmission mechanism of infectious diseases, predict the future transmission range and trend, and provide an important scientific basis for implementing scientific and effective prevention and control measures^10^. Therefore, this study aims to use mobile network data to construct a model of the spatial transmission pattern of New Coronary Pneumonia to provide decision support for relevant functional departments and make relevant policies more accurate and efficient, so as to guarantee the smooth operation of the economy and people's life to the maximum extent.

## Literature review

In recent years, scholars have studied the evolution of infectious disease transmission by constructing models such as SIR, SEIR, and SEIRD. Zhang studied the dynamics of the SEIR infectious disease model with saturated incidence and saturated treatment functions, giving the basic regeneration numbers that determine disease extinction and disease survival^11^. Affandi explored the formation of a modified malaria distribution model through a SIR modeling study. The malaria distribution model was used to analyze the infection rate in South Kalimantan for optimal control of malaria transmission in South Kalimantan^12^. Ferjouchia used the SEIRD model to provide a theoretical framework for the prediction of the ongoing epidemic of COVID-19 in Morocco, and the structure and parameters of the proposed model provided insights and ideas for the development of virus dynamics^13^. Side (2021) used the SEIR model to make predictions about the number of epileptic fevers in the city of Makassar using the number of people suffering from epileptic fevers in Haji Hospital in Makassar as auxiliary data. The results of the analysis indicate that epileptic fever is in a stable phase: the trend of epileptic fever in the city of Makassar, especially in the Tamarat subdistrict, is decreasing every year^14^. Serina introduced the SEIR model to simulate the spread of SARS-CoV-2 in the ten largest metropolitan areas in the United States and showed that a few "super-spreader" points of interest accounted for the majority of infections and that limiting the maximum occupancy of each point of interest was more effective than uniformly reducing mobility^15^.

The evolution of epidemic transmission has its characteristics, according to which reasonable interventions can effectively reduce the risk of epidemic transmission^16^. Direct attention should be paid to factors affecting their spread that may be underestimated, especially indoor spread and outdoor risk sources^17^. The contribution of important indoor factors such as ventilation systems to the spread of this virus needs to be carefully determined, and outdoor risk sources such as aerosol particles and particulate matter emitted during sewage treatment should also be examined, both of which may act as virus carriers, and the impact of environmental conditions on transmission should be considered when developing prevention and control measures. Kumar also considered the relationship between environmental factors and outbreaks, reviewing recent national and international studies on the influence of abiotic environmental factors such as climate, temperature, humidity, wind speed, air and water quality, solid surfaces, and frozen foods, as well as biological factors such as age, sex, gender, blood group, population density, and behavioral characteristics on virus transmission, persistence, and infectivity. In addition, virus transmission pathways that may pose a threat to population health are discussed^18^. Araya uses agent-based modeling to understand the potential impact of COVID-19 on a specific group of people, such as construction worker^19^. Kraemer suggest that travel restrictions are particularly useful in the early stages of an outbreak, as the outbreak is limited to one area as the primary source. However, once an outbreak becomes more widespread, travel restrictions may become less effective. The combination of interventions implemented in China was successful in mitigating the spread of COVID-19 and reducing local transmission^20^. Han conducted a multi-scale geographic analysis of the spread of Covid-19 in a dynamic network of policy influences and showed the effectiveness of import control policies when the domestic spread is at least partially suppressed, and that uncontrolled domestic spread exponentially magnifies the impact of imports. The study further demonstrates the effectiveness of transmission controls and travel restrictions both domestically and internationally, with domestic transmission interventions being more important than domestic travel flow controls^21^.

Academic research on human mobility suggests that cell phone data can help model the geographic spread of epidemics^22^. Frank found evidence of profound changes in the structure of mobility networks by analyzing the cell phone records of 43.6 million people in Germany. These changes are mainly due to a reduction in long-distance mobility, leading to a more clustered local network and thus a more grid-based system. Reduced global mobility during the lockdown may have slowed the spread of the disease in space^23^. Nuria discussed the value and contribution of mobile data in the analysis of COVID-19 control, which can not only enable stakeholders in various sectors to better understand the trend and geographical distribution of COVID-19 but also determine the key mechanisms and consequences of implementing different measures to control the spread of COVID-19. Real-time demographic and mobile data can also be used to achieve predictive capabilities and to evaluate interventions^24^. Seth used location data from a large sample of smartphones, nationally representative consumer preference surveys, and government statistics to measure the relative spread risk benefits and social costs of closing approximately 30 different location categories in the United States, providing data support for policymakers to develop prevention and control measures^25^. Based on national telecommunications data from Switzerland from 10 February to 26 April 2020, Persson quantified the extent to which mobility was reduced by five different policy measures (assembly bans, school closures, place closures, and border closures) to help public decision-makers assess the impact of policy measures targeting mobility behavior^26^. While strict social distancing measures have proven effective in slowing the COVID-19 pandemic, there could be a second wave as restrictions are lifted. Alberto integrated anonymous, geo-located mobility data with census and demographic data to construct a detailed Agent-based model of the spread of Severe acute respiratory syndrome Coronavirus 2 (SARS-CoV-2) in the Boston metropolitan area. Studies have found that a period of strict social distancing, followed by robust testing, contact tracing, and home isolation, can keep the disease within the capacity of the healthcare system while enabling economic activity to restart^27^.

In summary, the trend direction of the epidemic can be effectively determined based on the infectious disease model, and based on this, the principles of prevention and control are proposed, indicating the importance of prevention and control policies to reduce the continued spread of the epidemic. At the same time, the use of mobile network data provides a basis for the study of the spatial evolution characteristics of the epidemic and the formulation of prevention and control measures.

In this paper, by analyzing the epidemic data and transmission mechanism, combining the SEIR model with mobile network data, and based on the Safegraph website and officially published epidemic data, we simulated the trend of the number of crowd visits and the number of epidemic infections for different functional points of interest after the implementation of the reopening strategy, and accordingly judged the impact of the point of interest on the risk of epidemic development, and further discussed the period in which whether the reopening strategy could be adopted and the corresponding prevention and control measures were analyzed.

## Data and methods

### Data source

In this paper, the population movement data from March to May 2020 provided by Safegraph were adopted, and Python and visual studio code were used to build an infectious disease model covering the population movement data. The number of newly confirmed cases on that day can be predicted by taking into account the population flow and the changes in susceptible and latent populations. On this basis, the points of interest are divided into seven categories: restaurants (including ordinary restaurants and restaurants that only accept take-away packages), gyms, religious centers, convenience stores, large shopping malls, and pharmacies. And the impact of reopening strategies of different points of interest on the number of infections was simulated in the New York area as an example to provide a decision basis for reopening points of interest in different epidemic phases, so as to protect people's lives to the greatest extent possible while meeting their daily needs.

### Construction of spatial evolution model of COVID-19

#### Model description

To address the different transmission characteristics exhibited by Covid-19 in different countries and regions, this paper cites the infectious disease model constructed by Chang^12^. We also combine mobile network data with the SEIR model to specifically discuss the effect of dynamic population movement on the number of COVID-19 infections^28^. For each area, a personal mobile dichotomous network between CBG (census block group) and POI (point of interest) is constructed, where the weights of the edges between CBG and POI indicate the number of people between them at a given time.

**Figure 1:**
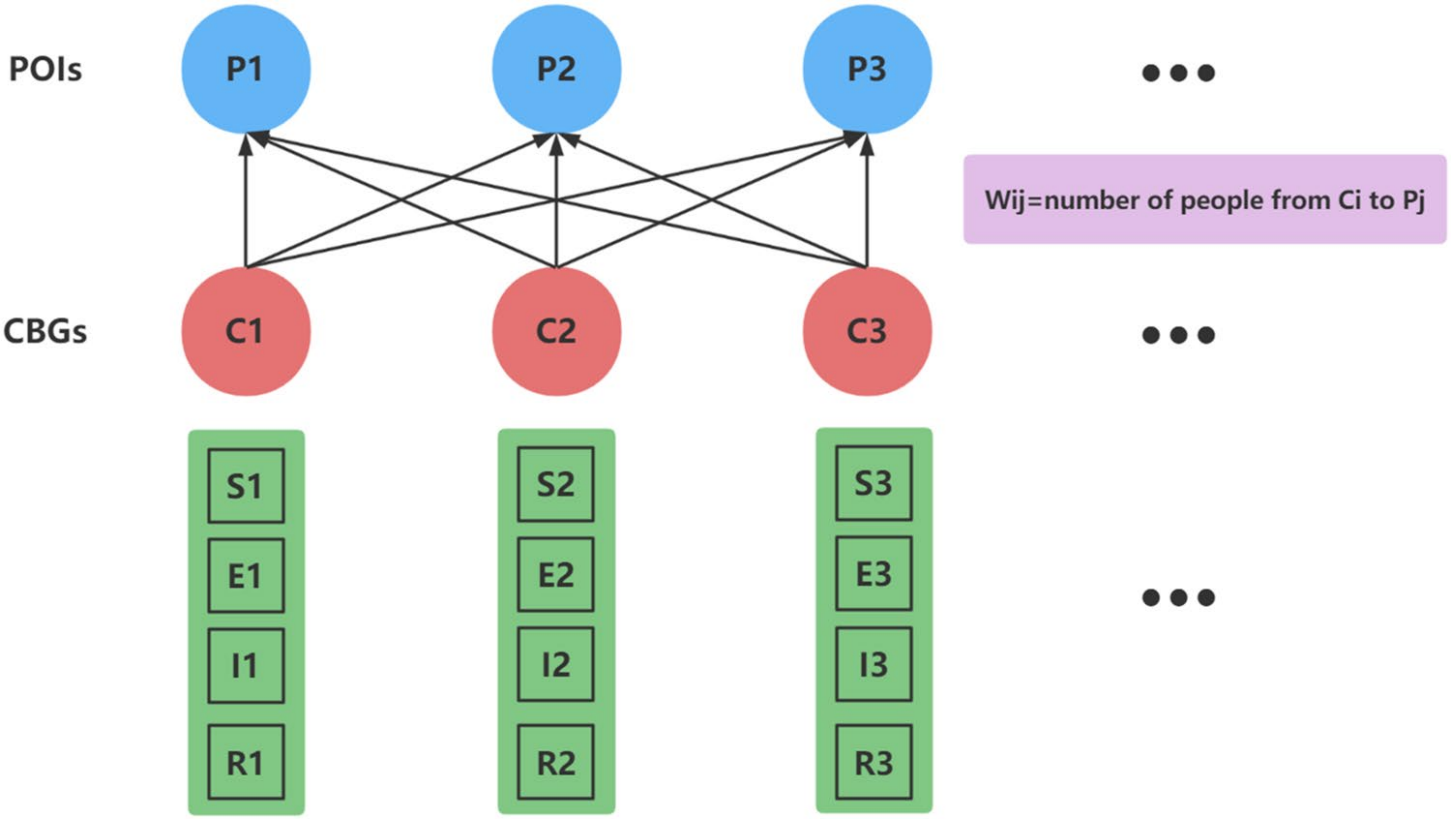
Diagram of SEIR mobile network model.

SEIR model has been widely used in the study of infectious diseases and has a good prediction effect. It divides the total population into four chambers, namely the susceptible population (S), the exposed population (E), the infected population (I), and the recovered population (R). Specifically, the individuals in chamber S are not infected but are easily infected. Individuals in chamber E are infected but not yet contagious. In chamber I the individuals are infected and contagious. The individuals in the R chamber have been cured or free of disease and are immune. A SEIR model is superimposed on each mobile network, where each CBG maintains its S (susceptible population), E (exposed population), I (infected population), and R (recovered population) status as shown in Fig. 1.

In this model, susceptible individuals are never infected but can be acquired through exposure to infectious individuals, which can occur at POI or CBG. Then they enter an exposure state during which they are infected but not yet infectious, with the rate of transition from exposure to infection inversely proportional to the mean incubation period. Finally, they are transformed into a cured population at a rate inversely proportional to the average duration of infection, and the cured population is not infectious and will not be reinfected in the short term.

In Fig. 1, each CBG (C1, C2, C3,…) has its own SEIR model, which contains four chambers, S^(t)^_ci_, E^(t)^_ci_, I^(t)^_ci_, and R^(t)^_ci_, representing the number of people in that CBG in four different states at time t, and N^(t)^_ci_ = S^(t)^_ci_ + E^(t)^_ci_ + I^(t)^_ci_ + R^(t)^_ci_. At time t, construct the following equations for the transitions between the states:

1 $${N}_{{S}_{ci}\to {E}_{ci}}^{(t)}\sim POIs\{\frac{{S}_{ci}^{(t)}}{{N}_{ci}}\sum_{j=1}^{n}{\lambda }_{pj}^{(t)}{w}_{ij}^{(t)}\}+Binom({S}_{ci}^{(t)},{\lambda }_{ci}^{(t)})$$

2 $${N}_{{E}_{ci}\to {I}_{ci}}^{(t)}\sim Binom({E}_{ci}^{(t)},\frac{1}{{\delta }_{E}})$$

3 $${N}_{{I}_{ci}\to {R}_{ci}}^{(t)}\sim Binom({I}_{ci}^{(t)},\frac{1}{{\delta }_{I}})$$

Among them, $\lambda_{pj}^{(t)}$: the underlying infection rate of POIpj at time t; $w_{ij}^{(t)}$: the number of population visits from CBGci to POIpj at time t; $\lambda_{ci}^{(t)}$: the underlying infection rate of CBGci at time t; $\delta_{E}$: average incubation period; $\delta_{I}$: average duration of transmission.

Modeling the infection rates of CBGci and POIpj at time t. Assume that at each hour, each susceptible individual in CBGci has the basic probability of being infected and transitioning to an exposed state as:

4 $$\lambda_{ci}^{(t)} \,{ = } \,\beta_{{{\text{base}}}} \frac{{I_{ci}^{(t)} }}{{N_{ci} }}$$

Then the infection rate of POIpj per hour is:

5 $$\lambda_{pj}^{(t)} = \beta_{pj}^{(t)} \left( {\frac{{I_{pj}^{(t)} }}{{V_{pj}^{(t)} }}} \right){ = }\mu b_{pj}^{2} \frac{{V_{pj}^{(t)} }}{{a_{pj} }}$$

Among them, $\beta_{{{\text{base}}}}$: basic transmission rate; $\beta_{pj}^{(t)}$: transmission rate of POIpj at time t; $V_{pj}^{(t)}$: the number of people present at each POIpj at time t; $\mu$: transmission constant in POIs; $a_{pj}$: physical area of POIpj; $b_{{{\text{pj}}}}$: percentage of time spent per hour by people visiting pj.

For the number of new confirmed cases per day for all CBGs, the following equation was constructed:

6 $$N_{{{\text{cases}}}}^{d} = r_{c} \sum\limits_{i = 1}^{m} {\sum\limits_{{\gamma = 24(d - 1) + 1 - \delta_{c} }}^{{24d - \delta_{c} }} {N_{{E_{ci} \to I_{ci} }}^{\gamma } } }$$

d: time (number of days); $\gamma$: time (number of hours); M: total number of CBG; $r_{c}$: percentage of detected cases to all cases; $\delta_{c}$: average period of confirmation.

#### Parameter setting

Based on the study of Covid-19 and the characteristics of the model established in this paper, the parameters and their taking values were set as shown in Table 1. In the initial state, the infected population and the recovered population are both 0. All infected persons appear in the chamber of the exposed population, and the initial exposed population ratio is set. So the values of each chamber in the initially state are obtained. The total population in each area can be obtained from the U.S. Census Bureau; the average incubation period of Covid-19 is 96 h, the average transmission period is 84 h, the average confirmation period is 168 h, and the average detection rate was 10%. The number of population visits from the census block group to the point of interest and the area of the point of interest were obtained from the SafeGraph data site; the initial exposed population ratio, the base transmission rate, and the transmission constant in POI_s_ were taken from the model test.

**Table 1:** SEIR model parameter meaning and the value.

Parameter	Description	Value
$a_{pj}$	Initially susceptible population of CBG_ci_	(1-$p_{0}$)$N_{ci}$
$$E_{ci}^{(0)}$$	Initially exposed population of CBG_ci_	$$p_{0}$$$$N_{ci}$$
$I_{ci}^{(0)}$	Initially infected population of CBG_ci_	0
$R_{ci}^{(0)}$	Initially recovered population of CBG_ci_	0
$N_{ci}$	Total population of CBG_ci_	See in^29^
$a_{pj}$	Average incubation period	96^30^
$\delta_{I}$	Average infectious period	84^31^
$\delta_{C}$	Average duration of diagnosis	168^32^
$r_{c}$	Percentage of detected cases to all cases	10%^33^
$p_{0}$	Initially exposed population ratio	10^–5^~10^–2^
$\beta_{{{\text{base}}}}$	Basic transmission rate	0.0012 ~ 0.024
$\mu$	Transmission constants in POI_s_	515 ~ 4886
$w_{ij}^{(t)}$	Number of population visits from CBG_ci_ to POI_pj_ at time t	SafeGraph
$a_{pj}$	Physical area of POI_pj_	SafeGraph

### Model construction of reopening strategy

The infectious disease model constructed in this paper combines mobile network data with the SEIR model to determine the impact of human mobility behavior on the spread of the New Coronary Pneumonia epidemic. Here, POIs are divided into seven categories: restaurants (subdivided into regular restaurants and take-out-only restaurants), gyms, religious activity centers, convenience stores, large shopping malls, and pharmacies. For each POI the impact of the reopening strategy is simulated from the beginning of March 2020 to the beginning of May 2020, and the following functional model is constructed:

7 $$h(t) = \left\{ {\begin{array}{*{20}c} t \\ {f(t)} \\ \end{array} \, \begin{array}{*{20}c} {if{\text{ t < T}}} \\ {otherwise} \\ \end{array} } \right.$$

Among them, f(t) = t mod 168. T is the last hour of observed mobility data, and the function keeps t constant if there is observed mobility data at time t; otherwise, it maps t to the corresponding hour of the first week of the simulation.

To simulate the reopening strategy, which will be uniformly reduced to a proportion $\eta$ of its original level, where $\eta \in [0,1]$. The access matrix is:

8 $$\tilde{W}_{\eta }^{(t)} = \left\{ {\begin{array}{*{20}c} {W^{h(t)} } \\ {\eta W^{h(t)} } \\ \end{array} \, \begin{array}{*{20}c} {if{\text{ t < }}\gamma } \\ {otherwise} \\ \end{array} } \right.$$

where $\gamma$ represents the first hour of reopening. The actual observed mobile network data is used if observed mobility data is available during time t; otherwise, a proportion $\eta$ is used to simulate the mobility level.

Next, the maximum occupancy rate M_pj_ of each POI_pj_ is estimated as its maximum access during the simulated period. Set $w_{ij}^{(t)}$ as the number of CBG_ci_ accessing POI_pj_ in time t, and $V_{pj}^{(t)}$ as the total number of people accessing POI_pj_ in that hour, i.e., $\sum\nolimits_{i} {w_{ij}^{(t)} }$. The access matrix $\tilde{W}_{\beta }^{(t)}$ is constructed to simulate the maximum value at the maximum occupancy rate $\beta$, where $\beta \in [0,1]$:

9 $$\tilde{W}_{ij\beta }^{(t)} = \left\{ {\begin{array}{*{20}c} {W_{ij}^{h(t)} } \\ {\frac{{\beta M_{pj} }}{{V_{pj}^{(t)} }}W_{ij}^{h(t)} } \\ \end{array} \begin{array}{*{20}c} {{\text{ if t < }}\gamma {\text{ or V}}_{pj}^{(t)} \le \beta M_{pj} } \\ {otherwise} \\ \end{array} } \right.$$

For each POI_pj_ and time t, the matrix remains constant when reopening has not yet started or when the total number of visits to POI_pj_ during time t is below the maximum allowed $\beta M_{pj}$. Otherwise, a scaling factor $\frac{{\beta M_{pj} }}{{V_{pj}^{(t)} }}$ is calculated to reduce the total number of visits to POI_pj_ during time t to the maximum allowed, and then all visits to pj for each CBG_ci_ are proportionally reduced.

### Methodology for risk classification of POI

In this paper, the points of interest are divided into three risk levels: low risk, medium risk, and high risk. The corresponding reopening strategy shall be implemented according to the risk degree of different regions. For the classification of risk levels, see the table below (For the assessment of risk levels, two or more of the classification standards shall be met).

### Ethical approval

This study was not required to obtain ethics approval since it uses publicly available data that contains no identifiable private information. There is not any human data or human is directly involved in study. The authors did not have access to any personally identifiable information or information that would link the data to individuals’ identities.

## Results

### Impact of reopening strategies on outbreak development

**Figure 2:**
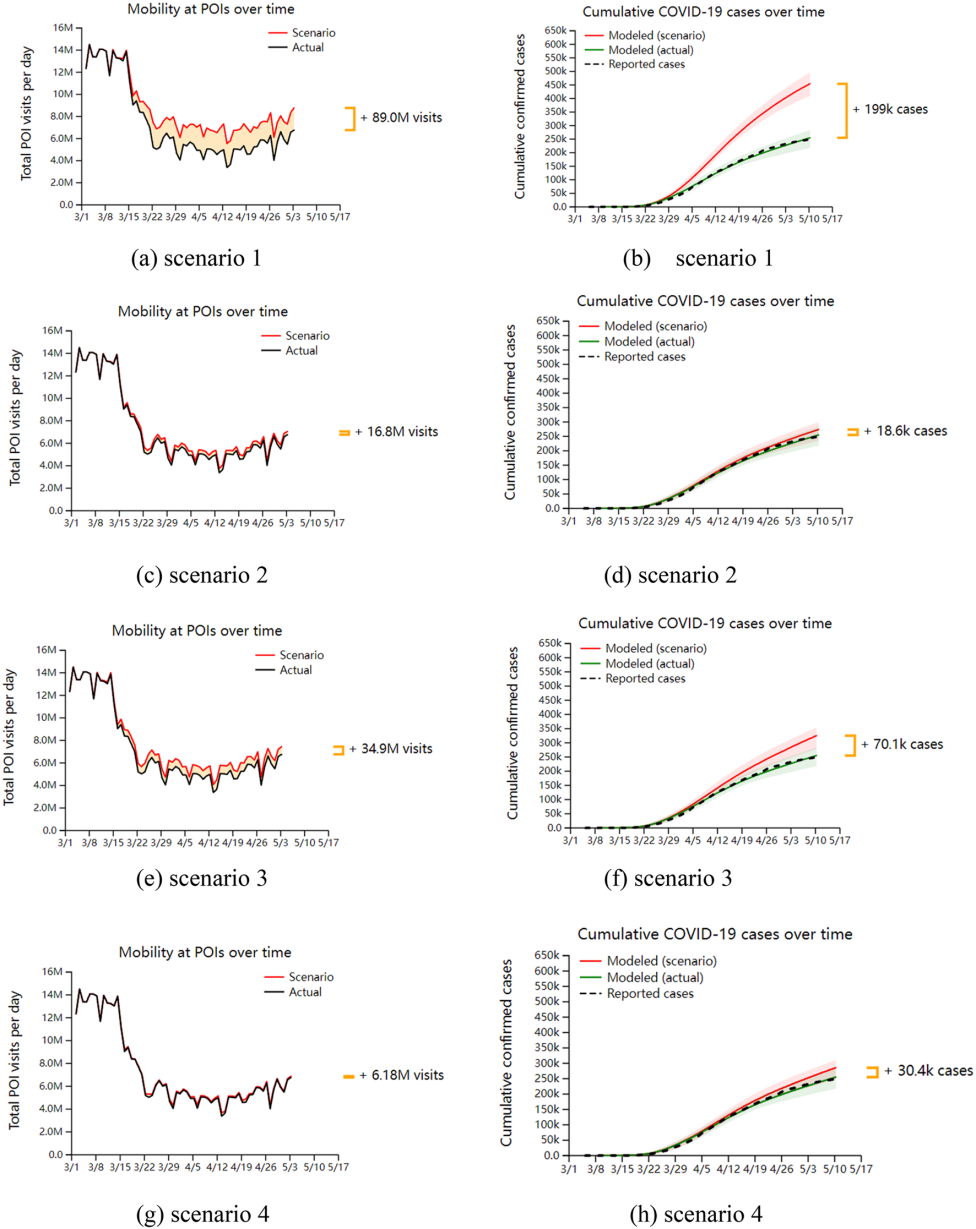

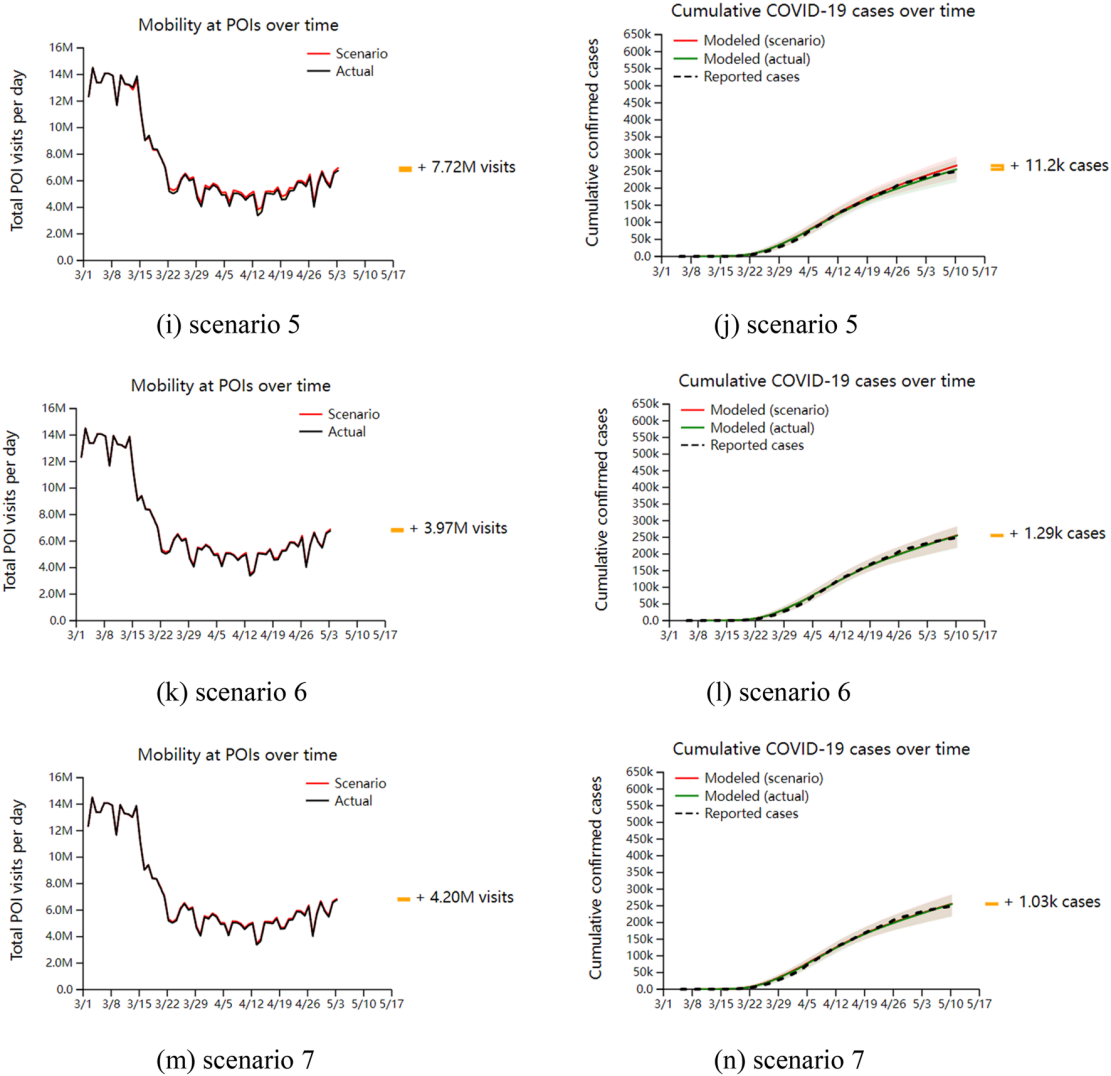
Simulation results under different scenarios. (**a**,**c**,**e**,**g**,**i**,**k**,**m**) is the change of the number of people visiting different interest points after the re-opening strategy is implemented. The black curve represents the number of people visiting under the situation of non-opening, and the red curve marks the number of people visiting after the re-opening strategy is implemented. (**b**,**d**,**f**,**h**,**j**,**l**,**n**) are the fitting results of the number of cases (green solid line) and the actual number of cases (black dotted line) obtained by the model when the reopening strategy was not implemented at each point of interest, as well as the changes of COVID-19 infection cases after the reopening strategy was implemented (red solid line).

Based on the research content of mobile network data overlaid in the SEIR model of infectious diseases, the simulation analysis is combined with the reality related to Covid-19. In this section, we take New York, one of the ten metropolitan areas in the United States, as an example, and analyze the impact of reopening different points of interest on the number of infections in the epidemic. The simulation’s initial time is set as March 1, 2020, and seven types of scenarios for the reopening strategy are set. Simulation results of reopening different points of interest are obtained, as shown in Fig. 2. Among them, scenario 1 is the opening of ordinary restaurants; Scenario 2 is the reopening of the restaurant that only accepts take-away packages; Scenario 3 is the reopening of the gym; Scenario 4 is the reopening of the religious activity center; Scenario 5 is the reopening of convenience stores; Scenario 6 is the reopening of the mall; Scenario 7 is the reopening of the pharmacy.

Based on the simulation results of the reopening strategy for the seven different categories of points of interest mentioned above, the following analyses were made. In all scenarios, from March 1 to early May 2020, the number of people visiting the points of interest after the implementation of the reopening strategy increased to different degrees, with the largest increase of 89 million people visiting general restaurants and the smallest increase of 3.97 million people visiting large shopping malls. At the same time, the number of infection cases also increased to different degrees after the implementation of the reopening strategy, among which the cumulative number of confirmed cases who visited general restaurants increased the most, 199,000, and the cumulative number of confirmed cases who visited pharmacies increased the least, 1030; the model fitted better with the real data when the reopening strategy was not implemented at the point of interest, which enhanced the credibility of the study results.

Scenario 1: After the general restaurant was reopened, there was a significant increase in the number of visits after 15 days, and the increase has been maintained in the subsequent time period, which shows that the demand for going to the general restaurant is the strongest after the reopening of points of interest with different functions. There was a significant increase in confirmed cases at about 30 days after the reopening, which is also consistent with the time period assumed in this paper from susceptible population to infected population for this length of time, and then the trend of increase continued to expand and the number of infections continued to climb.

Scenario 2: After reopening the take-out-only restaurant, the number of visitors also increased after 15 days, but the increase was much smaller than that of the regular restaurant, which shows that people's desire to go to the take-out-only restaurant was much smaller than that of the regular restaurant, and the increasing trend of confirmed cases was not obvious. Compared with the regular restaurant, the increase in the number of people visiting the take-out-only restaurant in the simulated time period was about 19% of their number and about 9% of their number of confirmed cases. The percentage increase in the number of confirmed cases was about one-half of the increase in the number of crowd visits, reflecting that the average infection rate in take-out-only restaurants after implementing the reopening strategy was lower than that in regular restaurants.

Scenario 3: After reopening the gym, consistent with Scenario 1, there was a significant increase in the number of visits after 15 days and a significant increase in the number of confirmed cases after 30 days, both of which were smaller in Increase than Scenario 1. The gym increased the number of crowd visits by about 39% and the number of confirmed cases by about 35% in the simulated time period compared to the regular restaurant, both of which had similar rates.

Scenario 4: There was no significant increase in the number of crowd visits after reopening the religious activity center, with a total of 6.18 million people moving through the site of interest during the simulation period, but there was a modest increase in the number of confirmed cases after 30 days. Compared to the general restaurant, the religious activity center increased the number of crowd visits by approximately 7% and the number of confirmed cases by approximately 15% during the simulation time period. The percentage of increase in the number of confirmed cases was approximately twice as high as the increase in the number of crowd visits, and the asymmetry between this rate also reflects the higher average infection rate in the religious activity centers after the implementation of the reopening strategy.

Scenario 5: There is no significant magnitude change in the curves for both the number of crowd visits and the number of confirmed cases after reopening the convenience store. The convenience store increases the number of crowd visits by approximately 9% and the number of confirmed cases by approximately 6% compared to the regular restaurant during the simulated time period, both at similar rates.

Scenario 6: After reopening the mega-mall, there is no significant magnitude change in the curves of both the number of crowd visits and the number of confirmed cases. Compared to the regular restaurant, the increase in the number of crowd visits in the mega-mall is about 4% and the increase in the number of confirmed cases is about 0.6% in the simulated time period. The percentage increase in the number of confirmed cases is about one-seventh of the increase in the number of crowd visits, demonstrating that the implementation of the reopening strategy resulted in a lower average infection rate in the mega-mall.

Scenario 7: After reopening the pharmacy, there is no significant magnitude change in the curves of both the number of crowd visits and the number of confirmed cases. Compared to the general restaurant, the increase in the number of crowd visits in the pharmacy is about 5% of its number and the increase in the number of confirmed cases is about 0.5% of its number during the simulated time period, and the percentage increase in the number of confirmed cases is about one-tenth of the increase in the number of crowd visits, reflecting that the implementation of the reopening strategy results in a pharmacy with the average infection rate was extremely low.

In summary, under the reopening strategy of seven different types of points of interest, the top three visits to the points of interest were general restaurants, gyms, and take-out-only restaurants, while the visits to religious activity centers, convenience stores, large shopping malls, and pharmacies did not differ significantly. Meanwhile, after the implementation of the reopening strategy, the top three sites with the highest average infection rates were religious activity centers, general restaurants, and gyms, while the sites with the lowest were large shopping malls and pharmacies. Therefore, the reopening of general restaurants, gyms, take-out-only restaurants, and religious activity centers had a significant impact on the development of Covid-19, while the reopening of convenience stores, large shopping malls, and pharmacies had little impact, for which targeted countermeasures should be taken in the development of outbreak prevention and control measures.

### Point-of-interest risk level classification

Based on the risk classification standard in Table 2, the seven points of interest covered in this paper can be divided into the following risk types as shown in the Table 3.

**Table 2:** Risk classification standard of POI.

Classification criteria	Risk level		
	Low risk	Medium risk	High risk
Increase in visits (V)	0 ≤ V < 10 million	10 ≤ V < 30 million	V ≥ 30 million
Increases in confirmed cases (C)	0 ≤ C < 10 thousand	10 ≤ C < 30 thousand	C ≥ 10 thousand
Total increase in confirmed cases as a proportion of total increase in visits (P)	0 ≤ P < 1‰	1‰ ≤ P < 2‰	P ≥ 2‰

**Table 3:** Risk classification of seven POIs.

Point of interest	Classification criteria			Comprehensive assessment of risk level
	V	C	P	
General restaurant	High risk	High risk	High risk	High risk
Take-out-only restaurant	Medium risk	Medium risk	Medium risk	Medium risk
Gym	High risk	High risk	High risk	High risk
Religious activity center	Low risk	High risk	High risk	High risk
Convenience store	Low risk	Medium risk	Medium risk	Medium risk
Large shopping mall	Low risk	Low risk	Low risk	Low risk
Pharmacy	Low risk	Low risk	Low risk	Low risk

The analysis shows that the points of interest with high risk are general restaurants, gyms, and religious activity centers, respectively. Medium-risk points of interest are restaurants that accept only pack-and-go, and convenience stores. Low-risk points of interest are large shopping malls and pharmacies, respectively.

According to the interest points of different risk levels, the corresponding minimum standards of preventive and control measures are established, and the preventive and control measures taken at each point should be no less than the minimum standards, and the specific minimum standards of preventive and control measures for each risk level are listed in the following Table 4.

**Table 4:** Minimum standards for prevention and control measures for points of interest at different risk levels.

Risk level	Minimum standard prevention and control measures
High risk	Restrict reopening completely. Points of interest that belong to a high risk level should not implement a reopening strategy until the outbreak is under control. The increasing number of confirmed cases will intensify the trend of the spread of the epidemic, making it more difficult to prevent and control the epidemic, and even leading to the loss of the effectiveness of the original epidemic efforts. Therefore, a reopening strategy should not be adopted for high-risk interests
Medium risk	Partial restriction on reopening. Points of interest that belong to the medium risk level also have an impact on the development of the epidemic to a certain extent. Therefore, the strategy of partial reopening can be adopted for points of interest that have an impact on people's daily production and living needs. For example, it is concentrated on a certain day of the week or a certain point of the day and not open the rest of the time; it is open according to the street, and each street has a fixed time in the month to open, etc. At the same time when people go to a point of interest with a medium risk level, they should take protective measures, show a health code and trip code, keep at least one-meter distance from others, and do not gather. Merchants should arrange staff to do a good job of measuring body temperature, registering basic information and maintaining the order of people entering the point of interest, ensuring that basic disinfection work has been completed when each person enters the store, and conducting extensive disinfection and sterilization work on the whole business premises after the end of the day's business
Low risk	Free and orderly opening in the same low-risk area locally. For low-risk points of interest in low-risk areas, people can move freely and orderly with basic protection. Merchants arrange for staff to take body temperature measurements, and people simply need to show their health code and not remove their masks in public places. In this case, all types of points of interest can be reopened with the completion of the minimum standards of prevention and control measures

## Limitation

In this study, we built an infectious disease model overlaid on population movement data, which tracked population movement and changes in susceptible and latent populations. There are seven categories of places involved in this paper, and the places involved are not comprehensive enough, and public places with a high pedestrian flow such as schools, cafes, and libraries can be included in future studies. At the same time, the development of public health emergencies is unpredictable, and more indicators need to be included for modeling for more accurate prediction, such as the amount number of supplies, medical situation (number of hospital beds, maximum treatment capacity, etc.), traffic conditions, the amount of time people spend in different locations, etc., in order to improve the fit between the real situation and the predicted situation. What’s more, different kinds of populations such as the fully vaccinated or those who have received booster doses are not distinguished, and these provide interesting directions for future work.

## Conclusion

By combining the SEIR model with mobile network data as described above, this paper simulates the trend of the number of crowd visits and the number of infected people in the epidemic for different functions of points of interest after the implementation of the reopening strategy, based on the Safegraph website and the officially published epidemic data, which accordingly determines the impact of the point of interest on the development of the epidemic and whether the reopening strategy can be adopted in that time period. The study showed that:

The number of visits to restaurants increased significantly after the implementation of the reopening strategy, with a greater increase in the number of visits to general restaurants and a smaller increase in the number of visits to restaurants that only accept packaged take-out; among all the points of interest, the number of confirmed cases increased the most among the population that visited the restaurants, with the number of confirmed cases in general restaurants increasing about ten times more than in restaurants that only accept packaged take-out. Therefore, the prevention and control policymaking should focus on taking measures for restaurants.

In addition to focusing on preventive and control measures for restaurants, attention should also be paid to gyms and religious activity centers. The former is the point of interest with the second highest number of crowd visits after the implementation of the reopening strategy, and the latter is the point of interest with the highest average infection rate after the implementation of the reopening strategy. Therefore, targeted prevention and control measures regarding these two points of interest should not be neglected.

Convenience stores, large shopping malls, and pharmacies were the three points of interest with a small increase in the number of visitors and a very low infection rate after the implementation of the reopening strategy. It is judged that these three points of interest can be reopened according to the current inherent prevention and control behaviors during the time period due to their special attributes and proper prevention and control.

## Discussion

Policymakers are struggling to identify a series of restrictions that will effectively contain the spread of COVID-19 without unduly depressing economic activity. Mobility data have enormous potential in public health decision-making. Several studies have outlined the different applications of mobile data in guiding and evaluating COVID-19 responses, but the lack of data-driven retrospective analysis is not ideal for guiding future decision-making^24,34^. Public health measures should be adjusted according to the movement behavior of different populations. When population movement behavior is linked to the likely course of epidemic transmission, then these measures can be better justified for decision-making.

In our study, we built an infectious disease model overlaid on population movement data, which tracked population movement and changes in susceptible and latent populations. This builds on previous research using aggregated or synthetic^35,36^ mobility data to model disease transmission; in addition, other studies have analyzed mobility data under the background of COVID-19, but there is no model for disease transmission^37,38^. By combining epidemiological models with these mobile networks, we can not only accurately fit the number of observed cases, but also conduct detailed analyses to inform measures for effective response to COVID-19.

Our findings can guide policymakers in rapidly evaluating competitive approaches to reopening. Key decisions not supported by strong situational epidemiological modeling in resource-constrained conditions, or over-emphasizing the result of data simulation while ignoring the policy-oriented guidance of the supporting data^39^, prompts calls for research to suggest data-driven decisions that can guide real-world work. Our model meets this demand, which simulates the changing trend of the number of people visiting and the number of infected people in different POIs after the implementation of the reopening strategy, divides the POIs into different risk levels, and proposes specific reopening strategies according to the risk levels of different regions.

According to the above analysis and the minimum re-opening strategy prevention and control criteria for different risk levels of points of interest in Fig. 2, the corresponding prevention and control measures are proposed for the seven types of points of interest covered in this paper.

The government can direct the opening sequence according to the overall increase in the number of visits to the point of interest and the number of infected cases. First open low-risk points of interest such as large shopping malls and pharmacies, which have less impact on the epidemic. The epidemic prevention and control policy should not be one-size-fits-all and should meet people's daily needs to the greatest extent possible without affecting the epidemic. From the simulation results, the number of visits to such places as large shopping malls and pharmacies does not increase much, and people visit such points of interest only when necessary; the number of confirmed cases also does not increase much, especially for such points of interest as pharmacies, which are particularly aware of their own epidemic prevention, and should be opened in time. As for the order of reopening the two types of points of interest, such as large shopping malls and pharmacies, pharmacies should be opened first, followed by large shopping malls.

The government could put restrictions on reopening only medium-risk sites like take-away restaurants and convenience stores. The reopening of medium-risk points of interest, such as take-out-only restaurants and convenience stores, also had a certain degree of impact on the epidemic, so we should restrict the reopening of these points of interest while maximizing people's daily needs. For example, the government can mandate that people can only go to the nearest convenience store in their community, and the convenience stores are open during certain hours or that residents of a certain community can only go during certain hours to maximize the dispersal of people, while adopting online ordering and offline delivery services. Take-out-only restaurants can be reserved in advance, and people can go and pick them up on their own without gathering or staying which applies to rider delivery services as well.

Until the epidemic is brought under control, the government should restrict the reopening of high-risk places such as gyms and religious centers. From the above study, it was found that there was also a strong demand for gyms and therefore a higher increase in the number of infections. Since gyms are confined spaces and people do not take protective measures when exercising, which increases the risk of infection, the reopening of gyms should be restricted until the outbreak is controlled. The government and media can promote people to exercise at home, which can reduce the risk of infection while meeting people's demand for exercise and fitness; meanwhile, religious activity centers are the points of interest with the highest average level of infection after the reopening strategy is implemented, reflecting that activities with multiple people gathering can cause the scale of the epidemic to grow, so the reopening of gyms and religious activity centers should be eliminated until the epidemic is controlled.

The government could allow take-out restaurants to partially open before regular restaurants. The increase in the number of visits to restaurants after the reopening of the different types of points of interest is the most obvious one, so there is nothing wrong to strengthen the necessary control over them. The number of visits to general restaurants can be found to be exploding, and the number of infections is also increasing especially. Until the epidemic is under control, all general restaurants should be closed, not allowing customers to stay too long in restaurants. Take-out-only restaurants are open to satisfy people's need to go to restaurants and reduce the number of infections.

The government should continue to promote the behavior of wearing protective masks and keeping social distance. According to the above simulation analysis, the increase in confirmed cases was mostly found in the dense points of interest where people did not wear protective masks. For example, people would gather together and take off their masks when eating in restaurants, working out and in gyms, and holding religious activities. Therefore, during the epidemic, the government should convey the idea of wearing protective masks outside and keeping a certain social distance, such as not removing masks outside and keeping a distance of more than one meter from people, in order to cut off the risk of virus transmission at the source and maximize the safety of people's lives.

## Data Availability

Mobile phone mobility data are freely available through the SafeGraph COVID-19 Data Consortium (https://www.safegraph.com/covid-19-data-consortium) case and death counts of New York city (https://github.com/nytimes/covid-19-data) is publicly available. Some of the estimated models are available at the website (http://covid-mobility.stanford.edu). All python code is accessible from the corresponding author.
